# The Roles of FOXO1 in Periodontal Homeostasis and Disease

**DOI:** 10.1155/2021/5557095

**Published:** 2021-03-30

**Authors:** Liang Ren, Jing Yang, Jun Wang, Xuedong Zhou, Chengcheng Liu

**Affiliations:** State Key Laboratory of Oral Diseases, National Clinical Research Center for Oral Diseases, West China Hospital of Stomatology, Sichuan University, Chengdu, Sichuan, China

## Abstract

Periodontitis is an oral chronic inflammatory disease that is initiated by periodontal microbial communities and requires disruption of the homeostatic responses. The prevalence of periodontal disease increases with age; more than 70% of adults 65 years and older have periodontal disease. A pathogenic microbial community is required for initiating periodontal disease. Dysbiotic immune-inflammatory response and bone remodeling are characteristics of periodontitis. The transcription factor forkhead box protein O1 (FOXO1) is a key regulator of a number of cellular processes, including cell survival and differentiation, immune status, reactive oxygen species (ROS) scavenging, and apoptosis. Although accumulating evidence indicates that FOXO1 activity can be induced by periodontal pathogens, the roles of FOXO1 in periodontal homeostasis and disease have not been well documented. The present review summarizes how the FOXO1 signaling axis can regulate periodontal bacteria-epithelial interactions, immune-inflammatory response, bone remodeling, and wound healing.

## 1. Introduction

Periodontitis is a chronic inflammatory disease of the tooth-supporting tissues that is initiated by pathogenic microbial communities and results in progressive destruction of the periodontal tissues, including the gingiva, periodontal ligaments, and alveolar bone [1, 2]. Severe periodontitis is the 6th most prevalent disease worldwide [3]. Epidemiological evidence shows that the occurrence and severity of periodontitis increase with age [4]. While early studies indicated that a triadic group of microbes comprising *Porphyromonas gingivalis*, *Treponema denticola*, and *Tannerella forsythia* was the causative agent of periodontitis, more recent microbiome studies have discovered greater complexity to the etiology of periodontitis [5]. Emerging data show that complex microbial communities are the fundamental etiological agent, and periodontitis results from polymicrobial synergy within these communities which incites dysbiotic host responses [6, 7]. Colonization by keystone pathogens (e.g., *P. gingivalis*) can initiate a transition of the periodontal microbial community from a commensal microbiota to a dysbiotic microbiota, triggering host immune responses and facilitating pathobiont persistence in the local environment, further dysregulating the host immune-inflammatory state [1, 2]. Moreover, microbial dysbiosis and inflammation can reinforce each other in a reciprocating feedforward loop, leading to periodontal tissue breakdown [8]. For instance, colonization by *P. gingivalis* stimulates host cells to release various proinflammatory cytokines, such as interleukin-1*β* (IL-1*β*) and tumor necrosis factor-*α* (TNF-*α*), and recruits neutrophils to the site of infection [9]. Neutrophils can also induce the generation of ROS via the respiratory burst. At low concentrations, ROS are part of the host defense against infection [10]. Tamaki et al. found that the levels of reactive oxygen metabolites in the serum were positively correlated with immunoglobulin G antibodies against specific periodontal pathogens, including *P. gingivalis*, *Aggregatibacter actinomycetemcomitans*, and *Prevotella intermedia* [11].

Although a dysbiotic microbial community is required for initiation of periodontal disease, it should be noted that the deleterious effects of the host response to the microbial challenge, rather than the direct toxic role of microbiota, are the main cause of periodontal damage [8]. The gingival epithelium that lines the gingival crevice forms a barrier between colonizing bacteria and gingival tissues and together with antimicrobial proteins, e.g., defensins and antimicrobial peptides (AMPs), provides the first line of defense against invading periodontal pathogens [12]. Once this barrier is overcome, periodontal tissue destruction and bone resorption are the primary outcome of interactions between the microbiota and immune cells, including phagocytes, natural killer (NK) cells, dendritic cells (DCs), T cells, and B cells [13]. The cytokine system is a key modulator in the process. For instance, the well-established proinflammatory cytokines from IL-1, IL-6, and TNF families can exaggerate periodontal inflammatory responses and lead to tissue degradation. A persisting inflammatory environment may ultimately disrupt bone homeostasis. In particular, cytokines such as IL-1*β*, TNF, and IL-17 can stimulate the expression of the receptor of nuclear factor-*κ*B ligand (RANKL), thus inducing the maturation and activation of osteoclasts [14]. Therefore, a homeostatic balance between immune-inflammatory responses and antimicrobial activities as well as a balance between osteoblasts and osteoclasts is required for periodontal health. Numerous clinical periodontal reconstructive surgeries have been attempted to restore such lost tissues [15], and these surgical procedures can lead to different patterns of healing. Nevertheless, epithelization of the wound and wound stability are indispensable for the establishment of a new connective tissue attachment to a root surface [16].

The forkhead box O (FOXO) transcription factors regulate many facets of the cellular physiological process, such as oxidative stress response, apoptosis, cell cycle regulation, and cell survival and differentiation [17]. The FOXO family has four members in humans, including FOXO1, FOXO3, FOXO4, and FOXO6. FOXO1 is normally expressed in insulin-responsive tissues and organs, such as the liver, skeletal muscle, and adipose tissue [18], and has been extensively studied since it was first identified in alveolar rhabdomyosarcomas [19]. A potential role for FOXO1 in periodontal homeostasis and dysbiosis is emerging. To contribute to the understanding of this issue, the present review focuses on the involvement of FOXO1 in regulating periodontal bacteria-epithelial interactions, immune response, bone remodeling, and wound healing.

## 2. Regulation of FOXO1 Activity and Expression

**Figure 1:**
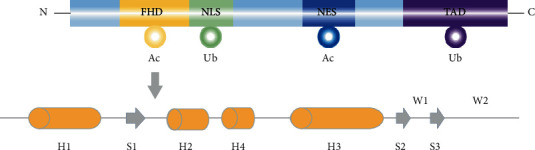
The structure of FOXO1. From the N terminus to the C terminus, FOXO1 contains a forkhead DNA-binding domain (FHD), a nuclear localization signal (NLS), a nuclear export sequence (NES), and a transactivation domain (TAD). Ac: acetylation; Ub: ubiquitination. A compact *α*/*β* fold contains four *α* helices (H1-H4), three *β* strands (S1-S3), and two wings (W1 and W2).

FOXO1 is considered a master control switch for multiple signals that enable an organism to maintain tissue homeostasis during stress [20]. The transcriptional activity of FOXO1 is regulated through a number of posttranslational modifications (PTMs), including phosphorylation, acetylation, ubiquitination, methylation, and O-GlcNAcylation. These PTMs affect FOXO1 subcellular localization, stability, and activity as a transcriptional regulator. FOXO1 along with other forkhead box O transcription factors (FOXO3, FOXO4, and FOXO6) shares a highly conserved 110-amino-acid DNA-binding domain, also known as the forkhead box or winged helix domain. These proteins also share a compact *α*/*β* fold that consistently contains four *α* helices (H1-H4), three *β* strands (S1-S3), and two wings (W1 and W2) (Figure 1). The regions showing the highest sequence conservation include the N-terminal region surrounding the first AKT/protein kinase (PKB) phosphorylation site, the forkhead DNA-binding domain (DBD), the region containing the nuclear localization signal (NLS), and part of the C-terminal transactivation domain [21]. Several sites for posttranslational modifications are located within or near the FOXO DBD, thus enabling the regulation of the interaction of FOXO with DNA, either directly or through protein-protein interactions [21–23].

Shuttling of FOXO1 between the nucleus and cytoplasm requires protein phosphorylation within several domains, and these are regulated by distinct signal transduction pathways, including the phosphatidylinositol 3-kinase/protein kinase B (PI3K/AKT) pathway, the mitogen-activated protein kinase/extracellular regulated protein kinase (MAPK/ERK) pathway, and the c-Jun N-terminal kinase (JNK) pathway [24, 25].  Table 1 provides a summary of known FOXO1 phosphorylation sites. Active PKB translocates to the nucleus and phosphorylates FOXO1 at three conserved residues, resulting in increased binding of FOXO1 to the regulator 14-3-3 and cytoplasmic localization of both proteins [20, 26]. Following cellular stress, particularly when high levels of ROS are generated, JNK becomes activated and phosphorylates FOXO1. This causes FOXO1 to translocate in the opposite direction, enter the nucleus, and display increased transcriptional activity [27].

Acetylation has also been demonstrated to regulate FOXO1 activity. The cyclic-AMP responsive element binding- (CREB-) binding protein (CBP), histone acetyltransferase paralogue p300, and p300/CBP-associated factor (PCAF) can acetylate lysine residues located in the forkhead domain [28, 29]. Conversely, enzymes of the sirtuin (Sirt) family catalyse NAD^+^-dependent deacetylation of FOXO1. Seven lysine residues (K245, K248, K262, K265, K274, K294, and K559) have been established as acetylation sites in FOXO1 [22, 28]. The acetylation of FOXO1 has been shown to result in both activation and inhibition of its transcriptional activity, depending on the cell types used and the FOXO1 target genes [30–32]. In most studies, deacetylation contributes to elevated FOXO1 activity and its transduction from the cytoplasm to the nucleus [31]. In addition, the above-mentioned lysine residues in FOXO1 can be ubiquitinated by S-phase kinase-associated protein 2 (Skp2).

The expression of FOXO1 genes is regulated in response to multiple physiological cues and pathological stimuli, such as oxidative stress and hormonal factors. E2 promoter binding factor 1 (E2F-1), p300, and forkhead box protein C1 (FOXC1) play critical roles in regulating FOXO1 gene transcription [33]. Additionally, numerous microRNAs and the RNA-binding protein, HuR, have been described as posttranscriptional regulation mechanisms of FOXO1 [34].

## 3. The Role of FOXO1 in Periodontal Homeostasis and Disease

As a critical signaling integrator, activated FOXO1 participates in maintenance of homeostasis and adaptation to environmental changes, properties that are important in periodontal health. Disruption of physiologic FOXO1 signaling, therefore, has potential relevance for periodontal dysbiosis.

### 3.1. FOXO1 in Bacteria-Epithelial Interactions

**Figure 2:**
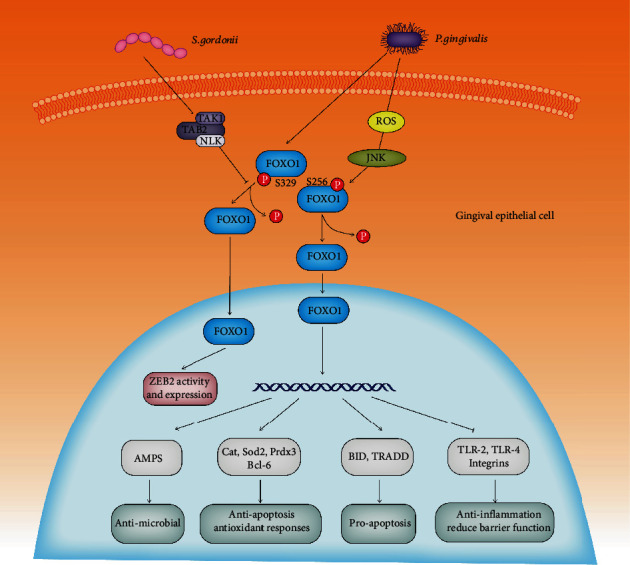
Regulation of FOXO1 activity by periodontal microbiota in gingival epithelial cells. FOXO1 (S256) can be dephosphorylated by *P. gingivalis* via ROS-JNK, promoting the nuclear localization of FOXO1. In the nucleus, FOXO1 mediates gene expression related to antimicrobial, antiapoptosis (Bcl-6), antioxidant response (Cat, Sod2, and Prdx3), proapoptosis (BID, TRADD), anti-inflammation (TLR-2 and TLR4), and epithelial barrier function. FOXO1 (S329) can also be dephosphorylated during *P. gingivalis* infection, resulting in the activation of ZEB2, and this process can be inhibited by the negative role of TAK1-NLK pathway, which is activated by *S. gordonii*.

The epithelium acts as a physical barrier to prevent the entry of bacteria into the underlying connective tissues [35]. Dysbiotic bacteria-epithelial interactions disrupt the integrity of the periodontal tissues. Previous studies have demonstrated that *P. gingivalis* can induce increased expression and activity of FOXO1 in gingival epithelial cells [36, 37]. The upregulation and activation of FOXO1 lead to the production of AMPs by the epithelium and to the elevated levels of antioxidant genes (e.g., CAT, SOD2, and PRDX3), apoptotic genes (BCL-6, BID, and TRADD), toll-like receptors (TLR-2 and TLR-4), and integrins, which together contribute to the control of potentially pathogenic bacteria [36, 37]. Interestingly, regulation of apoptotic genes by FOXO1 depends on *P. gingivalis* exposure time. Short-time exposure increases the antiapoptotic gene (BCL-6), while long-term exposure reduces proapoptotic genes (BID and TRADD) [36, 37]. In addition, upregulation of zinc-finger E-box-binding homeobox 2 (ZEB2) by *P. gingivalis* in gingival epithelial cells is also mediated through the pathway involving FOXO1. The homeostatic commensal *Streptococcus gordonii* can suppress FOXO1 induction and antagonize ZEB2 induction by *P. gingivalis* via activating the TGF-beta-activated kinase 1-Nemo-like kinase (TAK1-NLK) pathway [38] (Figure 2). Collectively, these results suggest that FOXO1 comprises a component of host epithelial response to periodontal bacteria. However, activation mechanisms and biological impact on the epithelium remain to be understood in future studies. Additionally, the role of FOXO1 in organizing the epithelium response to the subgingival plaque *in vivo* is still missing.

### 3.2. FOXO1 in Immune-Inflammatory Responses

In the periodontally healthy state, host-bacteria interactions are balanced, and when homeostasis is disrupted, the innate and adaptive immune systems work in concert in recognition and disposal of the periodontal bacteria. The role of FOXO1 in the immune system, especially dendritic cells, T cells, and B cells, has been comprehensively reviewed [39]. Here, we mainly focus on the potential relevance of FOXO1 for periodontal homeostasis and disease.

#### 3.2.1. FOXO1 in Innate Immune Responses

The innate immune response to the invading bacteria is mediated mainly by phagocytes, NK cells, and DCs. After contact with phagocytes, initially neutrophils and later macrophages, bacteria generally are rapidly ingested and killed inside the cell. Some organisms are resistant to degradation within phagocytes, which cause the activation of NK cells. NK cells can also be activated by DCs [8].

**Figure 3:**
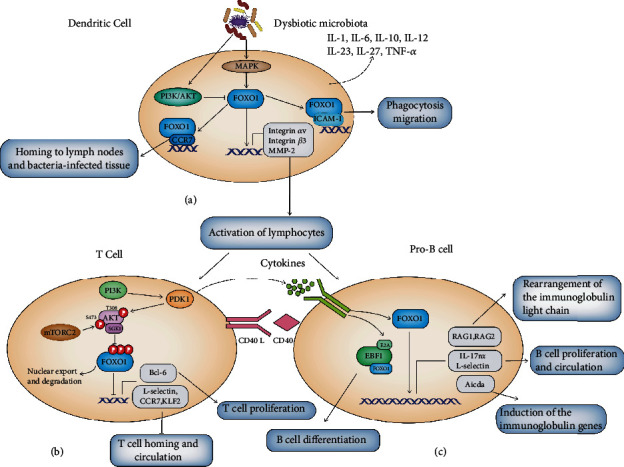
FOXO1 participates in function of DCs, T cells, and pro-B cells. (a) Dysbiotic microbiota stimulation initiates a signaling cascade to regulate the activity of FOXO1 via the MAPK and PI3K/AKT pathways. Activated FOXO1 can upregulate the transcription of CCR7, ICAM-1, integrin *α*v, integrin *β*3, and MMP-2, thus homing DCs to lymph nodes and bacteria-infected tissue, promoting phagocytosis migration and activation of lymphocytes. (b) FOXO1 associates with T cell proliferation, T cell homing, and circulation, which depend on counteracting the PI3K-, mTORC2-, and AKT-dependent negative regulation of FOXO1. (c) In pro-B cell, FOXO1 can be activated by various cytokines and participates in rearrangement of the immunoglobulin light chain, B cell proliferation and circulation, and B cell differentiation by promoting expression of key target genes, such as RAG1, RAG2, IL-17r*α*, L-selectin, Aicda, and EBF1.

In physiological conditions, neutrophils, which constitute ≥95% of total leukocytes in the gingival crevice, form a defense “wall” which protects the underlying tissues from periodontal bacteria [40, 41], and patients with neutrophil defects are more susceptible to periodontal disease [42]. Previous studies have provided initial evidence that FOXO1 may favor the survival and recruitment of neutrophils [43, 44]. For instance, Yang et al. found that FOXO1 is capable of forming a complex with myeloid cell leukemia-1 (MCL-1) and coordinate neutrophil survival [44]. In agreement, FOXO1 is also needed to mobilize neutrophils from the bone marrow to the vasculature and to recruit neutrophils to sites of bacterial infection [43]. Moreover, the potential role of FOXO1 in macrophage polarization has also been studied. Highly expressed FOXO1 was found in M2 macrophages, and M2-like macrophages show FOXO1 enrichment on the IL-10 promoter following lipopolysaccharide (LPS) treatment [45]. Further, both Sirtuin 3 (SIRT3) and TGF-*β*-mediated macrophage M2-like polarization can occur via FOXO1-dependent pathways [46, 47]. Taken together, it is possible that FOXO1 promotes macrophage polarization towards the M2-like phenotype, thus suppressing inflammation and facilitating wound repair. Interestingly, in high-glucose conditions, macrophages exhibit an inflammatory phenotype, which is possibly due to reduced binding of FOXO1 to the promoter region of IL-10 [45]. Conversely, it has also been reported that FOXO1 activation can abolish M2 macrophage polarization and induce proinflammatory cytokine IL-1*β* expression [48]. In particular, FOXO1 is capable of binding to the IL-1*β* promoter and enhancing IL-1*β* promoter activity [48]. RNAi-mediated FOXO1 knockdown results in abrogation of the FOXO1-mediated induction of IL-1*β* promoter activity in LPS-stimulated macrophages [48]. IL-1*β* is a multifunctional cytokine that not only directly affects the regulation of various genes that are characteristically expressed during inflammation but also indirectly affects the stimulation of various cells to produce connective tissue catabolic and bone-resorptive mediators [14, 49]. This cytokine is also involved in osteoclast formation and bone resorption by inducing RANKL [50]. Chen et al. have also reported that FOXO1 is indispensable for protease-activated receptor 2 (PAR2) promotion of M1 macrophage polarization [51]. Thus, under these experimental conditions, FOXO1 is indispensable for promoting M1 macrophage polarization. These apparently contradicting functions may be reconciled if the role of FOXO1 in macrophages depends on the conditions. More importantly, the role of FOXO1 in macrophages needs to be directly tested *in vivo*. Recently, FOXO1 has been defined as a negative regulator of NK cell maturation and effector function [52]. The LPS of *P. gingivalis* can promote the proliferation and activation of NK cells *in vivo*. In turn, the NK cells produce IFN-*γ*, which can activate macrophages and promote killing of phagocytosed bacteria [53]. Thus, it is tempting to speculate that FOXO1 may attenuate NK cell-mediated periodontal bacterial killing. However, the role of NK cells is complex and additional studies are still necessary. Additionally, DCs can induce a protective response through induction of Th2 lymphocytes [54]. However, they may also potentially enhance periodontal bone loss through upregulation of Th1 or Th17 responses [55]. The linkage between FOXO1 and DCs has been well studied and systematically reviewed by Graves et al. [56]. In brief, FOXO1 is activated in DCs and it is crucial for DC homing to lymph nodes, binding to lymphocytes and formation of an immune synapse which activates lymphocytes [57, 58]. FOXO1 nuclear localization and activity are induced by the MAPK pathway and inhibited by PI3K/AKT [56] (Figure 3(a)). The role of FOXO1 in periodontal homeostasis and dysbiosis via DCs is condition dependent. With lineage-specific FOXO1 deletion mice, Graves et al. have demonstrated that decreased FOXO1 reduces the recruitment of DCs to the gingiva and impairs the function of DCs both in normal and in aging mice. Specifically, FOXO1 deletion reduced migration of DCs to lymph nodes and decreased IL-12 production at mucosal surfaces [58]. Moreover, FOXO1 induces transcriptional activity and stimulates expression of the adhesion molecule intercellular cell adhesion molecule-1 (ICAM-1), integrins *α*v and *β*3, C-C chemokine receptor 7 (CCR7), and matrix metalloproteinase-2 (MMP-2), all of which are needed for the activity of DCs [57]. Interestingly, when challenged by oral infection, FOXO1 deletion reduced the adaptive immune response of DCs in normal mice. Aging is associated with decreased FOXO1; however, increased adaptive immune response was observed in aged mice compared with young mice, and the increase was reversed by FOXO1 deletion in DCs.

#### 3.2.2. FOXO1 in Adaptive Immune Responses

Adaptive immunity is thought to have evolved to provide a focused and intense defense against infections that overwhelm innate immune responses [59]. Usually, the failure of the innate immune response to control periodontal infection results in the recruitment of T cells and B cells to the periodontium. The presentation of bacteria or bacterial antigen captured by specialized antigen-presenting cells (APCs), such as macrophages and DCs, activates T cells and B cells. As specialized APCs, activated DCs produce various cytokines, including IL-1, IL-6, IL-10, IL-12, IL-23, IL-27, and TNF-*α*, which affect the activation and biological activity of other innate and adaptive immune cells [60] (Figure 3(a)). Thus, the positive regulation of FOXO1 in DCs as mentioned above may also influence this process.

Several studies have also revealed that FOXO1 participates in the differentiation and metabolism of T cells. FOXO1-deficient T cells stimulated by transforming growth factor-*β* (TGF-*β*) *in vitro* show compromised Treg cell differentiation. *In vivo*, T cell-specific FOXO1-deficient mice showed decreased frequency and number of thymic Tregs among CD4^+^ T cells [61]. One possible mechanism of FOXO1 guiding the differentiation of CD4^+^ T cells relates to the PI3K-mTORC2-AKT signaling pathway [61, 62] (Figure 3(b)). CD4^+^ T cells can activate phagocytes through the action of the CD40 ligand (CD40L) and IFN-*γ*, resulting in bacterial elimination and cytokine production. A higher proportion of Tregs has been observed in peripheral blood samples and periodontal tissue samples from chronic periodontitis patients compared to those from healthy individuals [29, 63]. Inhibition of Treg function in the periodontal tissue of mice results in increased alveolar bone loss and inflammatory cell migration, associated with decreased anti-inflammatory cytokine production along with increased inflammatory cytokine (IFN-*γ* and IFN-*α*) and RANKL production [64]. In this regard, FOXO1-mediated T cell differentiation is considered as a protective response against advanced infection [65, 66]. Otherwise, excessive T cell-mediated recruitment and activation of phagocytes and cytokine production are capable of causing tissue injury, such as vascular changes associated with inflammation, bone resorption, and the infiltration of neutrophils into affected tissues [62].

Another role of FOXO1 involves T cell-secreted cytokines and the interaction of CD40L on CD4^+^ T cells with CD40 on the B cell surface, which results in the activation of B cells (Figure 3(c)). FOXO1 has been identified by Dengler et al. as the master transcriptional regulator that orchestrates the differentiation, activation, and proliferation of B cells [67]. Specifically, FOXO1 is upregulated during the early pro-B cell stage, and a decrease in FOXO1 protein levels impairs several stages of B cell development through regulation of key target genes, such as IL-17 receptor alpha (IL-17r*α*), recombination-activating gene 1 (RAG1) and 2 (RAG2), L-selectin, Aicda, and early B cell factor (EBF1) [68, 69]. In activated B cells, FOXO proteins exert their effects via the upregulation of both proapoptotic genes (e.g., BIM and BCL-6) and antiproliferative genes (e.g., P21 and P27) [70, 71]. Optimal B cell proliferation requires PI3K-dependent inactivation of FOXO transcription factors [72]. Thus, FOXO1 may play key roles in regulating T and B cells in a highly cell- and context-specific manner.

Collectively, progress in the field of FOXO1 in immune regulation has revealed its versatile and condition-dependent functions for periodontal homeostasis and disease. Physiologically, FOXO1 seems to be critical for the recruitment of neutrophils, polarization of macrophages, homing and function of DCs, and differentiation of T cells and B cells. It may function to respond to environment changes and work to counteract the potential damage caused by high glucose, bacterial infection, and aging through regulating immune responses. It will be of interest to study the lineage-specific FOXO1 knockout model further to identify the role of FOXO1 in polarization of macrophages. The linkage between FOXO1 and DCs has been well studied by Graves et al. in periodontal tissues. However, there is still no direct evidence demonstrating the activation and function of FOXO1 in other immune cells for periodontal homeostasis and disease. In particular, further exploration of FOXO1 in immune responses under pathological conditions such as periodontitis and diabetes mellitus will be important to establish the full involvement of FOXO1.

### 3.3. FOXO1 in Alveolar Bone Remodeling

The alveolar bone is part of the maxilla and mandible that forms and supports the tooth socket. It develops around each tooth follicle during odontogenesis. As the permanent tooth root forms and the surrounding tissues develop and mature, alveolar bone merges with the basal bone [73]. In physiological conditions, the alveolar bone is in the process of continuous reconstruction. Bone deposition by osteoblasts and bone resorption by osteoclasts maintain a dynamic balance during tissue remodeling and renewal. When the concentration of inflammatory mediators in the gingival tissues reaches a threshold, the pathways that lead to bone resorption will be overactivated and bone loss will occur [8, 73]. Interestingly, recent studies have also demonstrated that osteoblast lineage cells are critical for periodontal bone resorption by increasing the number of osteoclasts as well as osteoclast activity [74].

#### 3.3.1. FOXO1 in Bone Deposition and Bone Coupling by Osteoblast Lineage Cells

FOXO1 is highly expressed in osteoblasts under physiological conditions [75]. Conditional deletion of FOXO1 in osteoblasts can cause a decrease in osteoblast numbers, bone formation rate, bone volume, and bone mineral density in the spine and femur of mice. Notably, the influence of FOXO1 as a regulator of bone mass is specific among all FOXO proteins [75–77] as FOXO1 is thought to positively regulate new bone formation in osteoblasts by favoring resistance to excessive levels of ROS and counteracting deleterious consequences of oxidative stress on the cells [75, 77, 78]. Specifically, deletion of FOXO1 in mouse osteoblasts results in decreased expression and activity of superoxide dismutase 2 (SOD2), accompanied by elevated levels of ROS and lipid peroxidation end products [77]. Moreover, supplying the antioxidant N-acetyl L-cysteine (NAC), which can normalize redox levels, leads to the phenotypic bone abnormalities of FOXO1 osteoblast knockout mice as mentioned above [77]. These effects of FOXO1 relate to its role in regulating the activity of signal transduction pathways activated by ROS, p53, and p66shc [77]. FOXO1 can reduce the activity of P53 by inhibiting the expression of P19ARF and P16, thus mediating ROS-induced antiproliferative actions [79]. Similarly, FOXO1 can also inhibit the activity of p66shc and influence proapoptotic action of ROS [80]. Deletion of FOXO1 in osteoblasts also compromises amino acid import and protein synthesis, thus resulting in decreased levels of glutathione (GSH) and in a subsequent increase in ROS. This is associated with FOXO1-ATF4 interaction [77]. Interestingly, under conditions of a strong host response induced by *P. gingivalis*, FOXO1 has been reported as a proapoptotic factor, which was sustained and highly activated by the acquired immune response, thus inducing increased apoptosis of osteoblast and reduced bone coupling [81].

#### 3.3.2. FOXO1 in Bone Resorption by Osteoclasts

Intracellular H_2_O_2_ accumulation is critical for the differentiation and survival of osteoclasts. As a sensor, mediator, and regulator of redox signaling, FOXO1 is elevated in conditions with high levels of bone resorption and has the ability to regulate the formation and activation of osteoclasts [27, 81, 82]. Bartell et al. found that long-term combined deletion of FOXO1, FOXO3, and FOXO4 decreases physical bone mass by increasing osteoclast numbers and activity [83]. Furthermore, FOXO1 suppressed bone resorption by attenuating H_2_O_2_ accumulation [83]. Consistent with this study, Tan et al. demonstrated that FOXO1 acts as a cell-autonomous inhibitor of osteoclast differentiation and activity, which is partially mediated by MYC suppression [84]. However, the regulation of osteoclast formation and activity is a complex process, which is affected by multiple factors, and moreover, the same factor may play different roles in this process. Therefore, studies on the role of FOXO1 in osteoclasts generate disparate results with different approaches. For instance, Wang et al. found that FOXO1 is a direct player in osteoclast formation and activity by mediating the action of RANKL on NFATc1 and several downstream effectors, including dendritic cell-specific transmembrane protein, ATP6vod2, cathepsin K, and integrin *α*v. Lineage-specific deletion of FOXO1 in osteoclast precursors (LyzM. Cre^+^FOXO1^L/L^) leads to reduced RANKL-induced osteoclast formation and osteoclast activity [85].

#### 3.3.3. FOXO1 in Modulating Osteoblast Differentiation

Osteoblast differentiation is controlled by various external signals that induce a cascade leading to terminal differentiation of osteoblasts from mesenchymal cells and osteoblastic precursors [86]. Accumulating evidence indicates the involvement of FOXO1 in osteoblast differentiation [75, 87, 88]. In physiological conditions, FOXO1 plays an important role in promoting osteoblast differentiation, maintaining normal erythropoiesis and hematopoietic stem cell quiescence and survival. Siqueira et al. studied the role of FOXO1 in modulating osteoblast differentiation by a system in which preosteoblastic cells undergo terminal differentiation [87]. They reported upregulation of FOXO1 mRNA levels and DNA binding activity in normal cells during formation of mineralizing nodules. Interestingly, overexpression of FOXO1 reduced MC3T3-E1 cell number and the number of proliferating cell nuclear antigen-positive cells. Teixeira et al. found similar results with FOXO1 expression and activity increasing in mouse bone marrow mesenchymal stem cells (BMSCs) [88]. FOXO1 can also affect mesenchymal cell differentiation into osteoblasts by directly interacting with the promoter of RUNX2 and increasing its expression, further confirming the function of FOXO1 in osteoblast differentiation [88]. In addition, conditional deletion of FOXO1 in developing mice results in excessive levels of ROS in the bone and increased osteoblast apoptosis and reduced number of osteoblasts [75].

Conversely, a series of findings reported by another team suggest that FOXO1 activation can eventually aggravate the effects of oxidative stress on the bone. Almeida et al. reported that oxidative stress promotes the association of FOXO1 with *β*-catenin, thus suppressing Wnt-/T cell factor-mediated transcription and osteoblast differentiation [89]. Later, Iyer et al. confirmed this finding in vivo with mice lacking FOXO1 in bipotential progenitors of osteoblast and adipocytes, which suggest that FOXO1 could attenuate Wnt signaling, thereby decreasing the number of matrix-synthesizing osteoblasts and amount of bone mass [90]. A possible explanation for these contradictory results is that FOXO1 is an early molecular regulator in promoting differentiation of mesenchymal cells and preosteoblastic cells into osteoblasts. Age-related increased oxidative stress may stimulate FOXO1, therefore determining the role of FOXO1 in osteoblast differentiation.

Given the pivotal role FOXO1 can play in new bone formation and bone coupling by osteoblast lineage cells, resorption of the mineral matrix by osteoclasts, and differentiation and proliferation of precursor cells, it is potentially a very relevant player in alveolar bone remodeling. Besides, ROS levels fluctuate significantly in different periodontal microenvironments; thus, the critical role for FOXO1 in bone remodeling also indicates that FOXO1 may be involved in alveolar bone remodeling by fighting against oxidative stress. However, significant gaps exist in demonstrating the expression and activity of FOXO1 in alveolar bone whether in physiological or pathological conditions. Further, there is a need to understand the precise mechanism of FOXO1 in the alveolar bone remodeling process.

### 3.4. FOXO1 as a Key Player in Periodontal Wound Healing

The keratinized epithelium of the gingival and sulcular epithelial tissues acts as a barrier against invasion of periodontal bacteria and their products and provides protection for the underlying periodontal connective tissue [12]. There is also an impermeable seal of junctional epithelium and connective tissue between the external environment and the internal parts of the body [12, 91]. When periodontal tissues are damaged, a sequentially phased wound healing response is initiated. This process usually consists of four steps: hemostasis, inflammation, proliferation and granulation, and finally maturation of renewal tissue for remodeling. Periodontal tissue wound healing is similar to the healing process of skin epithelium, which is a complex scenario that involves the tightly regulated coordination of resident cells in epithelial and connective tissues, as well as cytokines, growth factors, and extracellular matrices [91]. Furthermore, continuously elevated proinflammatory mediators may result in excessive formation of disorganized connective tissue matrices. In addition, systemic host factors such as diabetes mellitus influence on wound healing, and poorly controlled diabetics often have disordered wound healing [92].

Within hours of injury or surgery, epithelial cells of the basal layer proliferate and migrate through the fibrin clot and breach [91]. The role of FOXO transcription factors in epithelial wound healing has been reviewed [93, 94]. In brief, FOXO1 differentially regulates both normal and diabetic wound healing. In normal healing, FOXO1 promotes epithelial cell proliferation and migration by upregulating the expression of transforming growth factor-*β* (TGF-*β*) and its downstream targets such as integrins (integrin *α*3 and integrin *β*6) and matrix metalloproteinases (MMP-3 and MMP-9), as well as reducing oxidative stress [93, 95]. Recent evidence suggests that high levels of ROS and subsequent oxidative stress are key contributors to the development of periodontal diseases [96]. During the inflammatory stage of wound healing, neutrophils produce large amounts of ROS [97], which result in oxidative stress and subsequently increase apoptosis in the deepest area of sulcular pockets [98], causing further induction of proinflammatory cytokines and DNA damage [96]. It has been shown that elevated intracellular ROS increases nuclear localization and thus activity of FOXO1 through c-Jun-N-terminal kinase (JNK) signaling in gingival epithelial cells, which can induce the expression of genes that counter oxidative stress (CAT, SOD2, and PRDX3) and apoptosis (BCL-6) [37]. Thus, we speculate that FOXO1 is a positive regulator of periodontal wound healing in normal conditions. Another mechanism by which FOXO1 may be involved in gingival epithelial wound healing is via promoting angiogenesis. Deletion of FOXO1 in keratinocytes causes reduced endothelial cell proliferation and impaired angiogenesis. These effects correlate with the decreased expression of vascular endothelial growth factor A (VEGFA) [99]. In addition, decreased type I collagen density accompanied by reduced collagen fiber organization was found at the wound site in FOXO1^+/-^ mice [100]. Collagen, especially type I collagen, is the major structural protein for gingival connective tissue. It is also a key component in wound healing by providing a biologic scaffold for cellular activities such as cell attachment, migration, proliferation, and synthesis of a number of proteins. In contrast to the positive function of FOXO1 in wound healing, another study demonstrated the inhibitory role of FOXO1 in normal wound healing [101]. This study reported that acute knockdown of FOXO1 could promote early stage epithelial wound healing by increasing the expression of proteins critical for reepithelialization, including fibroblast growth factor 2 (FGF2), adipoq, Notch 1, and Myosin X (MYO10) [101]. The disparate results of FOXO1 in normal wound healing may be caused by different methods for FOXO1 knockdown as, for example, keratinocyte-specific FOXO1 deletion is more suitable for periodontal wound healing. In addition, it is generally accepted that poorly controlled diabetes has an adverse effect on periodontal wound healing, which is also partially mediated by FOXO1. In high-glucose conditions, FOXO1 is also activated which causes delayed wound healing by increasing expression of C-C chemokine ligand (CCL20) [102].

Altogether, the evidence suggests that the potential role of FOXO1 as a key cell regulator during periodontal wound healing may depend upon the specific microenvironment. In normal conditions, FOXO1 may function as a positive regulator via the following possible mechanisms: (1) improving cellular antioxidant capacity and suppression of apoptotic cell death, thus positively regulating proliferation and migration of epithelial cells; (2) promoting angiogenesis; and (3) inducing collagen synthesis. However, in high-glucose conditions, there is an opposite effect of FOXO1 through induction of inflammatory gene expression.

### 3.5. The Role of FOXO1 in Periodontal Tissue Homeostasis

It is clear from the studies *in vitro* and *in vivo*, FOXO1 is sensitive to the environmental changes (oxidative stress and glucose level), which are closely related to periodontal homeostasis maintenance. More interestingly, a recent study found that FOXO1 exerted antioxidative effort on protecting human periodontal ligament stem cells (hPDLSCs) from cellular oxidative damage and promoting osteogenic differentiation capacity of hPDLSCs in the inflammatory microenvironment [103]. Therefore, in the complex scenario of host-microbe interactions, immune response, bone remodeling, and wound healing associated with periodontal disease, FOXO1 might contribute to periodontal tissue homeostasis at multiple levels in a context- and condition-dependent manner. Generally, FOXO1 can be involved in the following processes: (1) protecting gingival epithelial cells from oxidative damage and antiapoptosis in response to periodontal bacterial challenge; (2) inducing recruitment of neutrophils, homing and function of DCs, and differentiation of T cells and B cells in physiological conditions; (3) promoting bone deposition by osteoblast and osteoblast differentiation; and (4) accelerating epithelial wound healing in normal conditions. Therefore, we hypothesized that in periodontitis, periodontal tissue damage caused by excess oxidative stress response, inflammatory immune response, and impaired osteogenesis can be accomplished by a decrease in the activity of FOXO1. However, how to use the FOXO1 transcription factor in potential therapeutics still needs further exploration.

## 4. Conclusions

Periodontitis is a chronic inflammatory disease that is characterized by destruction of the tooth-supporting structures, such as gingivae, periodontal ligaments, and the alveolar bone. FOXO1 is implicated in bacteria-epithelial interactions, immune status, bone remodeling, and wound healing, all of which have direct relevance for periodontal homeostasis and dysbiosis. These include improving cellular antioxidant capacity and suppression of apoptotic cell death; recruitment of neutrophils to sites of bacterial infection and DCs to the oral mucosal epithelium; regulation of macrophage polarization and NK cell maturation; the differentiation of adaptive immune cells, such as Tregs and B cells; modulation of bone deposition by osteoblasts and bone resorption by osteoclasts as well as osteoblast differentiation; and regulation of wound healing. Therefore, FOXO1 may mainly function as a homeostatic regulator which coordinates responses to environmental signals that disturb the periodontal homeostasis. However, direct evidence for the mechanism of FOXO1 action in periodontal tissues under both physiological and pathological conditions requires further study.

## Figures and Tables

**Table 1:** Phosphorylation sites in FOXO1 protein.

Kinases	Abbreviations	Sites phosphorylated in FOXO1	The role on FOXO1 activity	References
Protein kinase B	AKT	T24, S256, S319	Inactivation, nuclear exclusion	Guo et al. (1999); Rena et al. (1999)
c-Jun N-terminal kinase	JNK	S256	Activation, nuclear localization	Wang et al. [37]
Extracellular regulated protein kinase	ERK	S246, S284, S295, S326, S413, S415, S429, S467, S475	Enhanced interaction with other transcription factors suggested	Asada et al. (2007)
p38 mitogen-activated protein kinase	p38MAPK	S284, S295, S326, S467, S475	Enhanced interaction with other transcription factors hypothesized	Asada et al. (2007)
Cyclin-dependent kinase 1	CDK1	S249	Activation, nuclear localization	Yuan et al. (2008)
Cyclin-dependent kinase 2	CDK2	S249	Inactivation, nuclear exclusion (S249 phosphorylation verified, but no nuclear exclusion in some cells)	Huang et al. (2006); Yuan et al. (2008)
Recombinant dual-specificity tyrosine phosphorylation-regulated kinase 1	DYRK1	S329	Inactivation, nuclear exclusion	Woods et al. (2001)
Nemo-like kinase	NLK	S329	Inactivation, nuclear exclusion	Kim et al. (2010)
Casein kinase 1	CK1	S325	Inactivation, nuclear exclusion	Rena et al. (2002)
Mitogen-activated protein kinase-activated protein kinase 5	MK5	S215	Activation	Chow et al. (2013)
Protein kinase R-like endoplasmic reticulum kinase	PERK	S298	Activation, nuclear localization	Zhang et al. (2013)

## Data Availability

Data are available on request.
